# *Plasmodium knowlesi* in pig-tailed macaques: a potential new model for malaria vaccine research

**DOI:** 10.1186/s12936-023-04788-9

**Published:** 2023-12-13

**Authors:** Melanie J. Shears, Rebekah A. Reynolds, Caroline J. Duncombe, Felicia N. Watson, Weston J. Staubus, Chris Chavtur, Annette M. Seilie, Tuan M. Tran, Sumana Chakravarty, Stephen L. Hoffman, Sean C. Murphy

**Affiliations:** 1 Department of Laboratory Medicine and Pathology, University of Washington, 750 Republican Street, F870, Seattle, WA 98109 USA; 2 Center for Emerging and Re-Emerging Infectious Diseases, University of Washington, 750 Republican Street, Seattle, WA 98109 USA; 3 Washington National Primate Research Center, University of Washington, 1959 NE Pacific Street, Seattle, WA 98195 USA; 4 Sanaria, Inc., 9800 Medical Center Drive, Suite A209, Rockville, MD 20850 USA; 5 Division of Infectious Diseases, Department of Medicine, Indiana University School of Medicine, Indianapolis, IN 46202 USA; 6 Department of Microbiology, University of Washington, 750 Republican Street, F870, Seattle, WA 98109 USA

**Keywords:** Malaria, *Plasmodium knowlesi*, Sporozoite, Pig-tailed macaque, *Macaca nemestrina*

## Abstract

**Background:**

*Plasmodium knowlesi* is an established experimental model for basic and pre-clinical malaria vaccine research. Historically, rhesus macaques have been the most common host for malaria vaccine studies with *P. knowlesi* parasites. However, rhesus are not natural hosts for *P. knowlesi*, and there is interest in identifying alternative hosts for vaccine research. The study team previously reported that pig-tailed macaques (PTM), a natural host for *P. knowlesi*, could be challenged with cryopreserved *P. knowlesi* sporozoites (PkSPZ), with time to blood stage infection equivalent to in rhesus. Here, additional exploratory studies were performed to evaluate PTM as potential hosts for malaria vaccine studies. The aim was to further characterize the parasitological and veterinary health outcomes after PkSPZ challenge in this macaque species.

**Methods:**

Malaria-naïve PTM were intravenously challenged with 2.5 × 10^3^ PkSPZ and monitored for blood stage infection by *Plasmodium* 18S rRNA RT-PCR and thin blood smears. Disease signs were evaluated by daily observations, complete blood counts, serum chemistry tests, and veterinary examinations. After anti-malarial drug treatment, a subset of animals was re-challenged and monitored as above. Whole blood gene expression analysis was performed on selected animals to assess host response to infection.

**Results:**

In naïve animals, the kinetics of *P. knowlesi* blood stage replication was reproducible, with parasite burden rising linearly during an initial acute phase of infection from 6 to 11 days post-challenge, before plateauing and transitioning into a chronic low-grade infection. After re-challenge, infections were again reproducible, but with lower blood stage parasite densities. Clinical signs of disease were absent or mild and anti-malarial treatment was not needed until the pre-defined study day. Whole blood gene expression analysis identified immunological changes associated with acute and chronic phases of infection, and further differences between initial challenge versus re-challenge.

**Conclusions:**

The ability to challenge PTM with PkSPZ and achieve reliable blood stage infections indicate this model has significant potential for malaria vaccine studies. Blood stage *P. knowlesi* infection in PTM is characterized by low parasite burdens and a benign disease course, in contrast with the virulent *P. knowlesi* disease course commonly reported in rhesus macaques. These findings identify new opportunities for malaria vaccine research using this natural host-parasite combination.

**Graphical Abstract:**

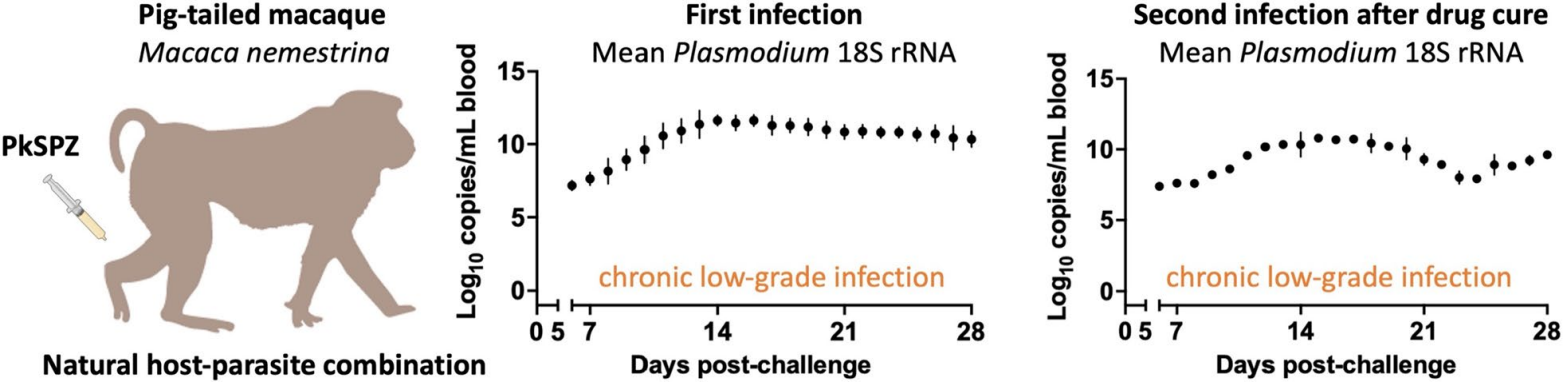

**Supplementary Information:**

The online version contains supplementary material available at 10.1186/s12936-023-04788-9.

## Background

*Plasmodium knowlesi* has a long history in malaria research that predates its recognition as a human parasite [1, 2]. These parasites are endemic to Southeast Asia, where they are commonly found in both long-tailed macaques (*Macaca fascicularis*) and pig-tailed macaques (*Macaca nemestrina*; abbreviated hereafter PTM) [3], and less frequently in other non-human primate (NHP) species [4]. As a research model, *P. knowlesi* sporozoites or blood stage parasites have been used to experimentally infect multiple NHP species, including rhesus macaques, long-tailed macaques, and other macaque species, as well as baboons, squirrel monkeys, and owl monkeys (reviewed in [5, 6]). These diverse experimental hosts display equally diverse disease outcomes during *P. knowlesi* blood stage infection, and correspondingly, different hosts have been used to study different aspects of parasite biology, disease pathogenesis, and immunity.

For vaccine research with *P. knowlesi*, the historical host of choice has been the rhesus macaque (*Macaca mulatta*) [6]. Rhesus macaques are extensively used in biomedical research, share ~ 93% of their genome with humans, and are considered excellent translational models for human immunity and disease [7]. *Plasmodium knowlesi* is highly infectious to rhesus macaques, making vaccine and challenge studies in this model a stringent test of malaria vaccine efficacy [8, 9]. Several highly impactful vaccine discoveries were made using this model (reviewed in [10]), and the wealth of prior literature has undoubtedly contributed to its popularity. However, this model has certain limitations. Firstly, *P. knowlesi* infection in rhesus macaques typically results in acute, severe, and potentially fatal, blood stage disease [10–21], necessitating rapid diagnosis and treatment of infected animals in order to comply with modern animal ethics guidelines [22]. Second, while rhesus macaques are highly susceptible to experimental *P. knowlesi* infection, they are not natural hosts for this parasite [6]. Thus, the model may not faithfully recapitulate all the features of a natural host-parasite combination that are relevant for vaccine studies.

COVID-19 research demands made rhesus macaques more expensive and difficult to source, and so inspired new interest in identifying alternative NHP hosts for malaria vaccine studies. The selection of an alternative host for vaccine studies should take several criteria into consideration, including how closely the model represents the human immune system, the availability of animals and immunological reagents, the reliability of infection after challenge, and the disease outcomes in that host. A further factor to consider may be whether the model is based on an experimental or natural host-parasite combination. Based on these criteria, the study team identified PTM a potentially promising alternative host for malaria vaccine studies. Pig-tailed macaques also share ~ 93% of their genome with humans [23], are bred for research [24], are considered excellent models of human immunity [25], and have a precedent for use in infectious disease studies [26–30]. Most importantly perhaps, PTM are natural hosts for *P. knowlesi*, and the disease course in wild animals is reportedly benign [31]. However, despite these many potential advantages, PTM have not previously been evaluated for use in basic or pre-clinical malaria research.

To evalute PTM as a potential model for malaria research, the study team therefore initiated a series of *P. knowlesi* challenge and infection studies at the Washington National Primate Research Center (WaNPRC). The team’s earlier report described preliminary data confirming that PTM could be challenged with cryopreserved *P. knowlesi* sporozoites (PkSPZ), with an equivalent time to blood stage infection as in rhesus macaques [32]. Here, additional exploratory studies were performed to further evaluate infection outcomes in this proposed new research model. The present pilot study aimed to address two of the criteria most pertinent to vaccine research: (1) the reliability of infection, and (2) the clinical characteristics of blood stage infection. To facilitate comparison with the published literature for the rhesus model, the team sought to define these infection outcomes in both malaria-naïve animals after initial challenge and in drug-cured malaria-experienced animals by re-challenge. The intent was to provide the first preliminary characterization of experimental *P. knowlesi* infection in the natural PTM host.

## Methods

### Study design and rationale

This study enrolled PTM (*Macaca nemestrina*) bred for research use at the WaNPRC. The WaNPRC colony founders were originally imported from Indonesia and Malaysia. All animals used in the study were malaria-naïve by virtue of being born in the United States, and all were tested for malaria at baseline and confirmed negative prior to enrolment. The study enrolled a total of n = 7 male and female PTM, aged three to 8 years, which were socially housed in pairs under standard conditions at the WaNPRC. The study was conducted in two cohorts. Cohort 1 consisted of n = 3 young adult males and was designed as a pilot study to assess the feasibility of PkSPZ infection. Cohort 2 consisted of n = 2 young adult males and n = 2 young adult females. Procedure details are provided in the study diagram (Additional file 1: Fig. S1). This follow-on study was designed to further characterize outcomes of PkSPZ infection in PTM and include animals of both biological sex per NIH policy [33]. For low-volume blood sampling to monitor *P. knowlesi* blood stage infection, animals were trained to enter a procedure cage for awake blood collection from the saphenous vein. For large-volume blood draws and other procedures that required handling, animals were fasted and sedated with intramuscular ketamine (10 mg/kg, Covetrus) per standard protocol. Some animals were given intramuscular ondansetron (0.5–1 mg/kg, Hikma) to reduce nausea, which is standard practice for sensitive animals at the WaNPRC. All procedures were conducted under an approved University of Washington Institutional Animal Care and Use Committee Protocol and in accordance with the NIH Guide for the Care and Use of Laboratory Animals.

### Cryopreserved sporozoites and intravenous challenge

This study used the *P. knowlesi* H strain, which was originally isolated from a natural human infection in Malaysia [15, 34]. Purified, cryopreserved, wild-type H strain PkSPZ were produced by Sanaria, Inc (Rockville, MD, USA). The PkSPZ are cryopreserved using patented methods. PkSPZ were shipped and stored on liquid nitrogen, and thawed and diluted according to standard Sanaria protocols. PkSPZ were administered to sedated animals intravenously in 1 mL of solution containing 1% human albumin (ALBURX, CSL Behring) as previously described [35]. The challenge dose for this study was 2.5 × 10^3^ PkSPZ. This dose was selected because prior experiments in rhesus macaques demonstrated that 2.5 × 10^3^ PkSPZ infects 100% of animals with highly reproducibile resulting blood stage parasite densities [32]. PkSPZ were vialled at 5 × 10^4^ PkSPZ per vial, so a single vial was used for all animals challenged at a given time.

### Monitoring of blood stage parasite burden

Daily monitoring of blood stage parasite burden was performed beginning 6 days post infection (p.i.), as this was the 1st day that parasites were expected to be in the peripheral blood [35]. For this, 200 µL of whole blood was collected into EDTA tubes and transported by courier at room temperature to the University of Washington Malaria Molecular Diagnostic Laboratory (MMDL). Blood collections typically occurred between 9:00 and 11:00AM, and samples were received at the MMDL by 12:00–1:00PM. Upon arrival, samples were processed in parallel for microscopic analysis and *Plasmodium* 18S rRNA RT-PCR by unblinded research technicians. Giemsa-stained thin blood smears were prepared according to standard protocols and examined under a 100X oil immersion objective on the day of collection. Fifty fields were viewed and the number and stages of the parasites recorded. This method has a limit of detection of ~ 100 parasites per µL of blood [36] and was used for same day monitoring of infection status. Animals were deemed to have a blood smear patent infection if two unambigious parasites were identified in fifty fields. For *Plasmodium* 18S rRNA RT-PCR, blood was spotted in 50 µL replicate spots onto Whatman Protein Saver cards (Cat #28170-017), air dried, and stored for later batch processing. Dried blood spots were excised by contact-free laser cutting into tubes [37], immersed in 2 mL of bioMérieux Nuclisens Lysis Buffer (bioMérieux, Cat #280134), and incubated at 55 ℃ for 30 min. RNA was extracted from 1 mL of lysate on the Abbott m2000sp platform (Abbott Molecular, Niles, IL) and 18S rRNA RT-PCR was performed as described on the Abbott m2000rt platform [35]. Animals were deemed to have RT-PCR positive infections if the endogenous control had a CT of greater than 25 and the Pan *Plasmodium* target detected greater than 5.17 log10 copies of 18S rRNA/rDNA per mL of blood, which is approximately equivalent to 20 parasites per mL of blood [38]. On selected study days, 50 µL of blood was mixed directly with 2 mL of NucliSens lysis buffer, then extracted and analysed as above. The 18S rRNA RT-PCR assay was used as the primary method to monitor parasite infection kinetics, since it could detect and quantify parasites at a far lower densities than possible by thin blood smears.

### Monitoring of disease signs

Daily observations of cohort 1 was performed beginning 6 days p.i. to assess outward signs of disease. More rigorous monitoring for disease signs was performed for cohort 2, with animals given a daily clinical score for alertness, respiration rate, food intake and fecal output. Blood from cohort 2 was sampled on selected study days for complete blood count, serum chemistry, C-reactive protein, and venous lactate tests. Tests were performed at baseline, day 0 prior to PkSPZ administration, and on days 4, 7, 10, 14, 21, and 28 p.i. The same schedule was followed after the second challenge, except for the final blood draw, which was performed at 29 days p.i. (Additional file 1: Fig. S1). Blood tests were performed by the University of Washington Department of Laboratory Medicine and Pathology Research Testing Service. Cohort 2 also underwent veterinary examinations and spleen ultrasound at baseline and 28–29 days after each challenge to qualitatively assess splenomegaly.

### Anti-malarial drug treatment

Anti-malarial drugs were provided orally in treats, unless otherwise noted, as this method is considered to be least intrusive for the animals. At the designated study day, cohort 1 was given oral chloroquine (20 mg/kg base) once per day for 3 days. Chloroquine was provided as a compounded suspension of chloroquine phosphate and was given in food treats or by oral gavage. For dosing in treats, the chloroquine suspension was mixed in 5 mL of a desirable food such as pudding or fruit puree in a paper cup. Animals typically consumed the treat under direct supervision, but in cases where they did not, consumption was confirmed by observing the empty paper cup. For oral gavage, when needed, the chloroquine suspension was delivered via an orogastric tube to sedated animals, followed by gavage of 10 mL of Ensure (Abbott Laboratories). Cohort 2 was given either oral chloroquine as above or oral Coartem (1 tablet per dose, each containing 20 mg artemether and 120 mg lumefantrine) twice per day for three days. Coartem tablets were crushed and dosed in treats as above. In response to a treatment failure with the above drug regimens, one animal in cohort 2 was additionally treated with intramuscular artesunate (8 mg/kg) for 4 days plus a 3-day course of oral chloroquine (20 mg/kg) every week for 3 weeks. Following treatment, blood was collected at least once per week to monitor for parasite clearance by 18S rRNA RT-PCR. Animals were deemed cured if they received negative RT-PCR test results for several consecutive weeks.

### Statistical analyses

As in previous studies [35], the number of animals per cohort was driven by budgetary constraints and sample sizes were relatively small. Descriptive statistics were used to summarize blood stage infection outcomes and haematological changes in each cohort, but in acknowledgement of the small sample size, statistical tests were not used to make comparisons between cohorts. All data should therefore be regarded as preliminary.

### Generation of gene expression data

Whole blood for gene expression analysis was collected on selected study days from cohort 2 animals into PAXgene tubes (BD #762165), transported at room temperature, and transferred to − 80 ℃ for storage. RNA extraction was performed using the Agencourt RNAdvance Cell V2 kit (Beckman Coulter #A47942). RNA quality was assessed using an Agilent TapeStation and yields were quantified using a Qubit Fluorometer. One hundred ng of RNA per sample was processed and loaded onto the NanoString NHP Immunology V2 Panel (NanoString #115000276) for targeted whole blood gene expression analysis.

### Gene expression analysis

The NanoString nCounter.RCC files were imported into nSolver Analysis Software 4.0 for review of quality control metrics, and the panel of housekeeping genes and positive controls was used to compute the normalization factor. Each expression set was then standardized to a calibration sample loaded onto each CodeSet in nSolver. Further data analysis was performed in RStudio version 2022.02.01+461 with R version 4.1.3. The log_2_ transformed normalized count matrix was evaluated for outliers using principal component analysis, and a single outlier was identified and removed since it did not cluster with any of the other samples, suggesting variation was technical rather than biological. Differential expression between timepoints was evaluated with a within-animal correlation to account for inter-individual baseline levels with time as the main effect. Differential expression testing was performed with limma [39] using an empirical Bayes moderation of the fitted linear model. Differentially expressed genes (DEGs) were defined as genes with a false-discovery rate (FDR) < 0.2, since when multiple comparisons are performed using the pre-selected NanoString panel, a less conservative method for gene significance levels is recommended. The union of DEGs identified by these criteria was visualized using UpSetR [40, 41]. Gene set enrichment analysis (GSEA) was performed against blood transcriptome modules (BTM) with fold-change as the ranking metric using fast GSEA [42, 43].

## Results

### Pig-tailed macaques can be reliably infected with purified cryopreserved PkSPZ

This study was designed to evaluate PTM as potential alternative hosts for malaria vaccine studies, with the aim of further characterizing the parasitological and veterinary health outcomes after infectious PkSPZ challenge. The study was conducted in two pilot cohorts. Cohort 1 consisted of three young adult males and was designed to assess the feasibility of PkSPZ challenge in PTM. This cohort was challenged intravenously with 2.5 × 10^3^ wild-type PkSPZ and monitored for blood stage infection by Giemsa-stained thin blood smears and *Plasmodium* 18S rRNA RT-PCR. Daily observations were used to assess disease signs. After a period of blood stage infection, cohort 1 animals were treated with anti-malarial drugs and returned to the colony. Preliminary data describing the time to blood stage infection following PkSPZ challenge in this cohort was reported previously [32]. This data is again presented here along with substantial new data from this cohort.

Cohort 2 consisted of two young adult males and two young adult females and was designed to further characterize outcomes of PkSPZ challenge in PTM. Cohort 2 was challenged with 2.5 × 10^3^ wild-type PkSPZ and monitored as above. This gave a combined total of seven naïve animals that were challenged with PkSPZ across cohorts. After drug treatment, cohort 2 animals were rested for around 2 months and considered for re-challenge. One female developed a recrudescent infection that required further drug treatment, so was excluded from re-challenge. Thus, three animals from this cohort went on to re-challenge. In addition to daily observations, cohort 2 animals were evaluated after challenge by daily clinical scoring, frequent complete blood count tests and serum chemistry tests, veterinary exams, and whole blood gene expression analysis.

**Fig. 1:**
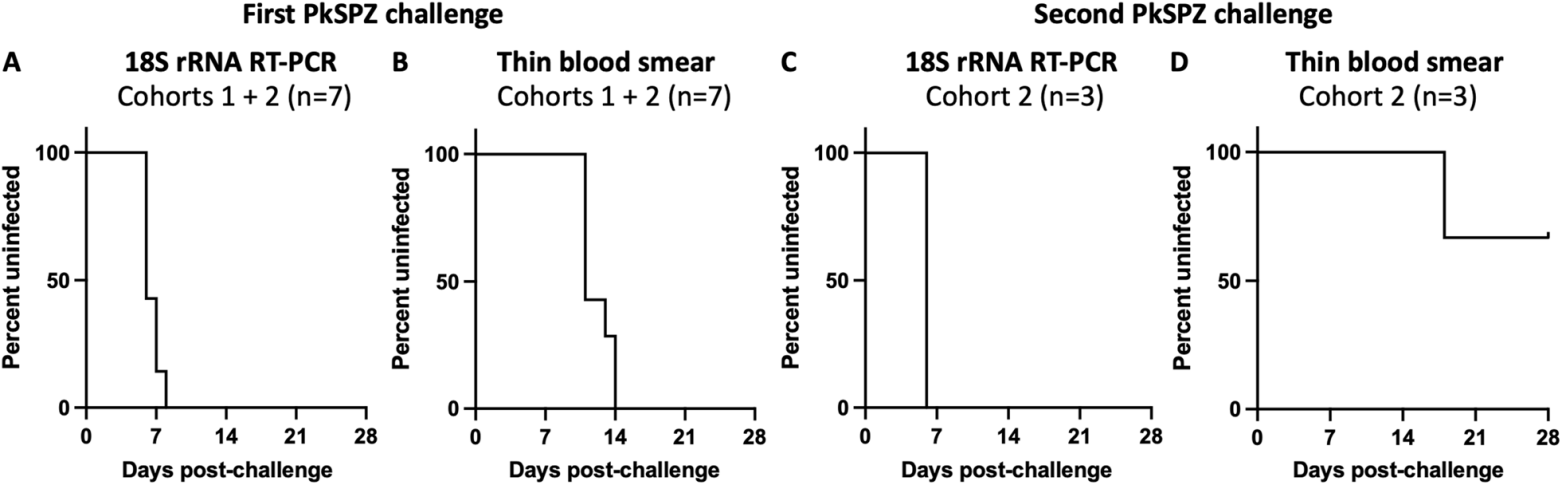
Time to detection of blood stage parasites after challenge of pig-tailed macaques with infectious *P. knowlesi* sporozoites (PkSPZ). **A**, **B** Naïve pig-tailed macaques from cohorts 1 and 2 were challenged intravenously with 2.5 × 10^3^ PkSPZ and monitored for time to blood stage infection by *Plasmodium* 18S rRNA RT-PCR and thin blood smear. **C**, **D** Approximately 2 months after drug-cure, cohort 2 animals were re-challenged and monitored as above. See Table 1 for data by individual animal

The time to first detection of blood stage parasites after PkSPZ challenge was consistent across cohorts (Table 1). All seven naïve animals became RT-PCR positive at 6–8 days post infection (p.i.) and blood smear positive at 11–14 days p.i. (Fig. 1A, B), consistent with RT-PCR having a much lower limit of detection than thin blood smears [36, 38]. These data were also comparable to the study team’s findings from naïve rhesus macaques challenged with the same PkSPZ dose [32]. After drug treatment, three of the cohort 2 animals successfully cleared their infections and went on to re-challenge. All three re-challenged animals became RT-PCR positive at 6 days p.i. (Fig. 1C). However, only one animal had sufficient parasitaemia to become blood smear positive and only did so at 18 days p.i. (Fig. 1D). Thus, naïve PTM reliably develop patent blood stage infections after PkSPZ challenge, with a time to first detection of blood stage parasites comparable to in rhesus macaques [32]. Then, upon re-challenge, all animals again reliably develop RT-PCR-detectable infections, but these typically remain below the limit of detection of thin blood smears.

**Table 1:** Time to detection of blood stage parasites after challenge of pig-tailed macaques with infectious *P. knowlesi* sporozoites (PkSPZ)

	Animal ID	Sex	PkSPZ challenge (days)		PkSPZ re-challenge (days)	
			18S rRNA RT-PCR	Thin blood smear	18S rRNA RT-PCR	Thin blood smear
Cohort 1	Z13228	M	7	11	No secondary challenge performed	
	Z16133	M	6	11		
	Z16318	M	6	11		
Cohort 2	Z14235	F	6	11	6	–
	Z16091	F	8	14	#	#
	Z17137	M	6	13	6	–
	Z18090	M	7	14	6	18*
Mean (days +/− SD)			6.6 +/− 0.8	12.1 +/− 1.5	6.0 +/− 0.0	18*

Data by individual animal. # = this animal was not re-challenged due to recrudescent infection. * = transient patency by thin blood smear. *M* male; *F* female, *SD* standard deviation

### Pig-tailed macaques spontaneously control *P. knowlesi* blood stage parasitaemia

**Fig. 2:**
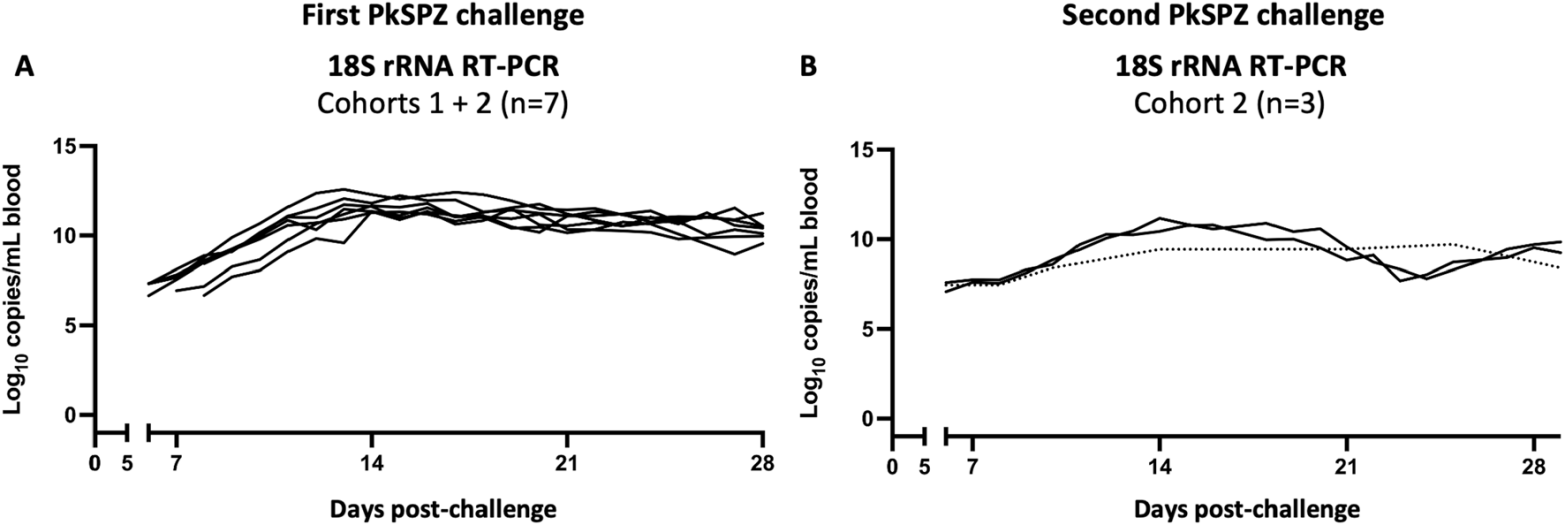
Kinetics of blood stage infection after challenge of pig-tailed macaques with infectious *P. knowlesi* sporozoites (PkSPZ). **A** Naïve pig-tailed macaques from cohorts 1 and 2 were monitored daily during blood stage infection to assess parasite burden by *Plasmodium* 18S rRNA RT-PCR. **B** Cohort 2 animals were monitored during their second blood stage infection as above. Data shown for each individual animal. Dotted line: fewer datapoints for this animal. See Graphical Abstract for the same data presented as mean ± standard deviation

Data from the daily RT-PCR monitoring was next used to evaluate the kinetics of blood stage infection in PTM after PkSPZ challenge. The kinetics of blood stage parasite replication in animals after challenge was highly reproducible across cohorts. In all seven naïve animals, *Plasmodium* 18S rRNA copy numbers rose linearly from days 6–11 p.i. during an initial acute infection phase, before copy numbers plateaued in a subsequent chronic infection phase (Fig. 2A). Concurrent analysis of blood smears indicated that 18S rRNA copy numbers plateaued around when animals became blood smear positive (Table 1). Indeed, positive blood smears contained very few parasites, suggesting parasitaemia was only just above the limit of detection of thin blood smears, which is between 0.01% and 0.001% parasitaemia [36]. Correspondingly, at no time did animals approach the treatment threshold of 1% parasitaemia, and anti-malarial treatment was only administered at the pre-defined study day at 28 days p.i. Thus, after PkSPZ challenge, naïve PTM show an initial linear increase in blood stage parasite burden, but later spontaneously control parasitaemia to develop chronic low-grade infections. This ability of PTM to limit blood stage replication contrasts with the uncontrolled parasitaemia typically reported for *P. knowlesi* in rhesus macaques [5, 44], and is instead more similar to that described in long-tailed macaques [10, 45–49].

Following re-challenge of cohort 2 animals, the kinetics of blood stage parasite replication by RT-PCR again appeared to be reproducible. However, one animal refused non-sedated blood draws after re-challenge, and data was limited for that individual animal. With this caveat, there again appeared to be a trend for 18S rRNA copy numbers to rise from 6–11 days p.i., before plateauing or decreasing (Fig. 2B). As noted above, concurrent evaluation of blood smears revealed that only one animal became transiently thin blood smear positive after re-challenge. Thus, re-challenged PTM again spontaneously control parasitaemia and develop chronic low-grade infections, and may limit *P. knowlesi* blood stage parasite replication to a greater extent than after initial challenge. The ability for PTM to achieve greater control of parasitaemia after re-challenge is consistent with data from both rhesus [14, 16, 17] and long-tailed macaques [10]. Further, it suggests that *P. knowlesi* infection induces functional immune responses in PTM, consistent with the findings from studies in these other hosts [10, 14, 16, 17].

### Pig-tailed macaques show a benign disease course during *P. knowlesi* infection

Daily observations and clinical scoring were used to assess the disease course in PTM after PkSPZ challenge. Cohort 1 animals were assessed only by daily observations, but no outward signs of disease or distress were noted. Cohort 2 animals were assessed by daily observations and daily clinical scoring for alertness, respiration rate, food intake and fecal output. The animals again showed no outward signs of disease, and no changes in clinical score that correlated with infection. Thus, PTM show an apparently benign disease course during *P. knowlesi* blood stage infection, consistent with reports from infected animals in the wild [31]. This outwardly asymptomatic course in PTM contrasts with the severe disease course typically reported for *P. knowlesi* in rhesus macaques [11], and is instead more similar to the disease course described in long-tailed macaques [10, 11, 46, 49].

The health of cohort 2 was additionally assessed by veterinary examinations, which were conducted at baseline and 28–29 days after challenge or re-challenge. These exams identified no remarkable findings aside from palpable enlargement of the spleen. After the initial challenge, three of the four animals had palpably enlarged spleens at 28 days p.i. Ultrasound confirmed changes in spleen shape relative to baseline in all four animals and increased echogenicity in two animals. After re-challenge, all animals had mild palpable splenomegaly at 29 days p.i. Ultrasound confirmed mild changes in spleen size or shape in two animals and a minor change in echogenicity in one animal. Therefore, all animals had evidence of splenomegaly after sustained periods of blood stage infection. These findings are consistent with prior reports of splenic involvement during *P. knowlesi* blood stage infection in both rhesus [11, 20, 50] and long-tailed macaques [47].

**Fig. 3:**
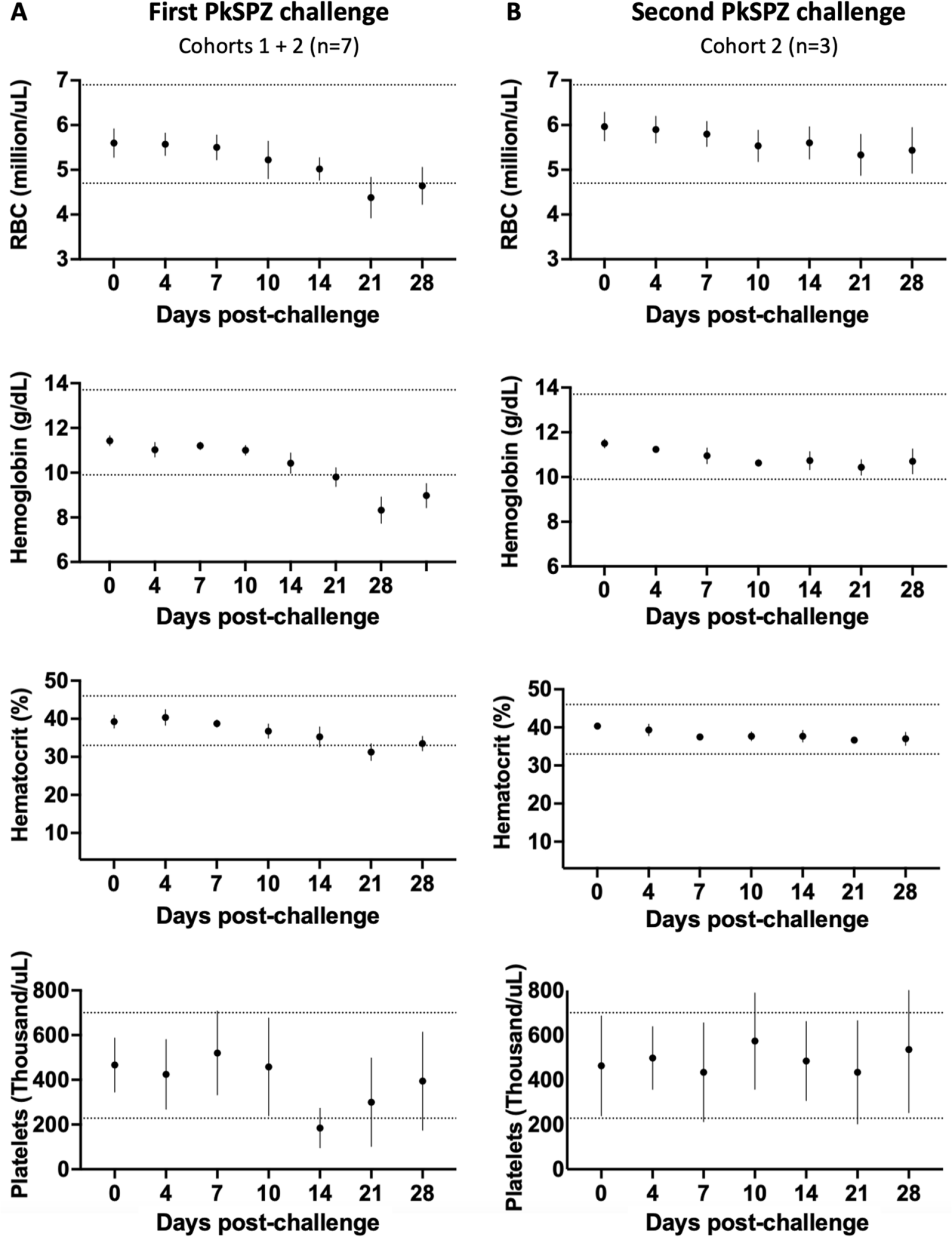
Complete blood count data for *P. knowlesi* infected pig-tailed macaques. **A** Naïve *P. knowlesi*-infected pig-tailed macaques develop anaemia and thrombocytopenia during their first blood stage infection. **B** *P. knowlesi*-infected pig-tailed macaques do not develop anaemia or thrombocytopenia during their second blood stage infection. Dotted lines show pig-tailed macaque complete blood count reference ranges at the Washington National Primate Research Center. Mean ± standard deviation

The disease course in cohort 2 animals was further monitored using frequent blood draws for haematology and serum chemistry tests, which included a panel of 18 analytes such as liver enzymes alanine aminotransferase (ALT) and aspartate aminotransferase (AST). No remarkable changes were seen in serum chemistry analytes at any point in the study. However, following initial challenge, all animals showed evidence of anaemia and thrombocytopenia, with decreases in red blood cell count, haematocrit, haemoglobin, and platelet count observed starting from 7 to 10 days p.i. (Fig. 3A). The anaemia appeared to be transient and mild, similar to previous reports of *P. knowlesi* in long-tailed macaques [47, 49]. Intriguingly, animals did not develop anaemia or thrombocytopenia after re-challenge, with red blood cell counts, haematocrit, haemoglobin, and platelet counts remaining almost exclusively within the normal reference ranges (Fig. 3B). Thus, malaria-naïve PTM develop anaemia and thrombocytopenia during their first *P. knowlesi* blood stage infection but develop resilience to these haematological changes upon subsequent infection.

### Transcriptional profiling of *P. knowlesi* infected pig-tailed macaques

The discovery that PTM can maintain *P. knowlesi* blood stage infection for many weeks, develop only mild haematological changes during their first infection, and go on to develop clinical resilience upon repeat infection are somewhat unique and distinguishing features of the model. To further explore how the PTM host responds to infection, whole blood gene expression analysis was performed for cohort 2 animals using the NanoString nCounter NHP Immunology panel. This panel includes 770 pre-selected immune response genes. Samples for this analysis were selected based on the *Plasmodium* 18S rRNA RT-PCR data (Fig. 2) and were intended to evaluate acute and chronic blood stage infection timepoints. For the first challenge, blood was sampled at baseline (first baseline), at day 10 p.i. (first acute), and day 28 p.i. (first chronic). For the animals that went on to re-challenge, blood was sampled immediately before re-challenge (second baseline), on day 10 p.i. (second acute), and day 29 p.i. (second chronic). These above shorthand timepoint designations are used hereafter.

**Fig. 4:**
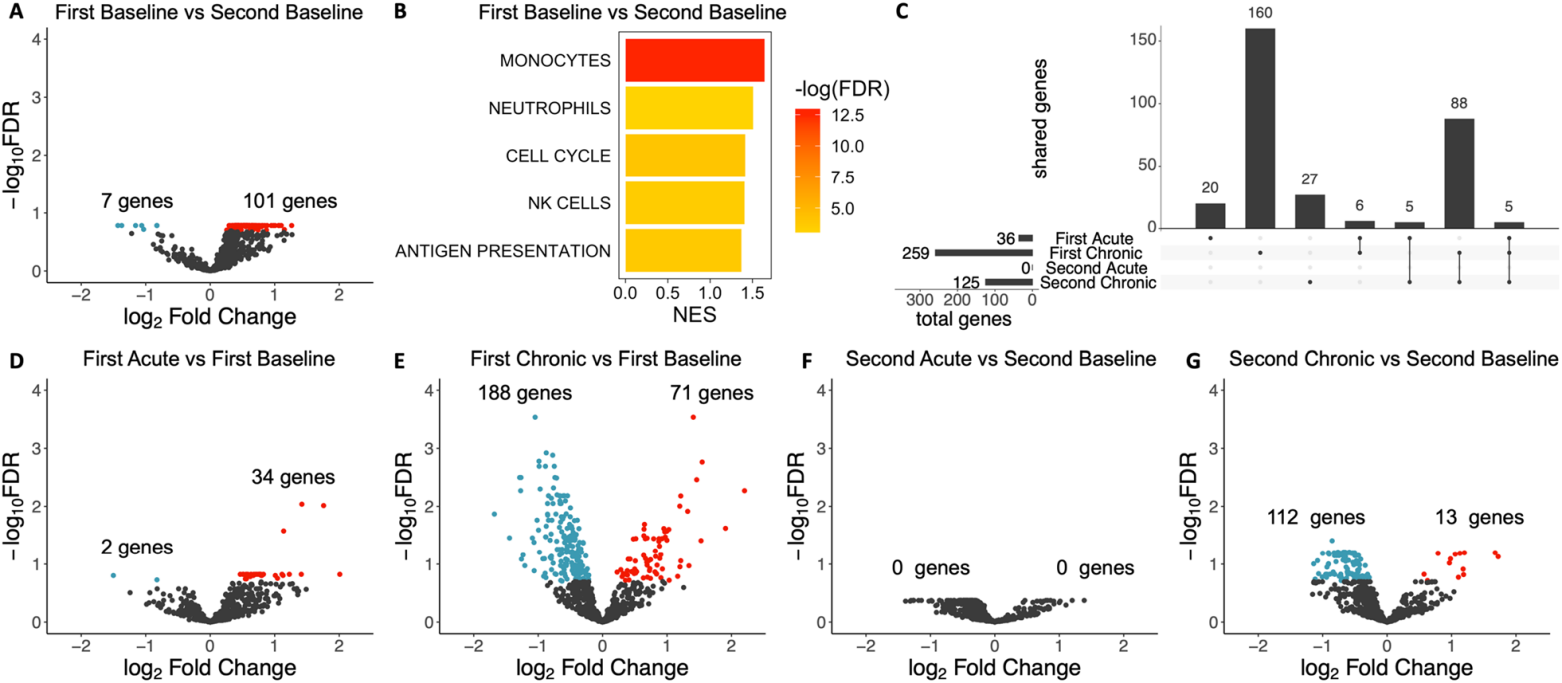
Overall trends in whole blood gene expression analysis of *P. knowlesi* infected pig-tailed macaques. **A** Volcano plot for first baseline versus second baseline. All volcano plots based on a False Discovery Rate (FDR) < 0.2, with upregulated genes shown in red and downregulated genes in blue. The number of Differentially Expressed Genes (DEGs) are indicated in each plot. **B** Gene Set Enrichment Analysis (GSEA) for blood transcription modules enriched in first baseline versus second baseline. GSEA based on an FDR of 0.05. NES, normalized enrichment score. **C** UpSet plot showing total DEGs for each infection timepoint versus the relevant baseline, and shared DEGs between infection timepoints. **D**–**G** Volcano plot for each infection timepoint versus the relevant baseline. All samples from cohort 2 animals

First, principal component analysis was performed on all genes in the panel, which showed that gene expression clustered by individual NHP, confirming that individual biological variation existed across NHPs enrolled in the study (Additional file 1: Fig. S2A). To assess overall variation in the gene expression data, pairwise contrasts between timepoints were used to identify differentially expressed genes (DEGs). Principal component analysis of the most variably expressed genes revealed that samples broadly clustered according to timepoint, suggesting that infection status was a driver of variation in the expression data (Additional file 1: Fig. S2B). Evaluating each pairwise contrast, an immediately notable finding was that the baseline samples differed substantially, with 108 DEGs identified between the first and second baselines (Fig. 4A). To investigate immunological processes associated with these DEGs, Gene Set Enrichment Analysis (GSEA) was performed using Blood Transcription Modules [42]. This analysis identified significant upregulation of monocyte, neutrophil, cell cycle, natural killer (NK) cell and antigen presentation modules at the second baseline relative to the first (Fig. 4B). Thus, even 2 months after drug treatment*, P. knowlesi* blood stage infection induced lasting immune system changes in this host, consistent with data from human malaria infection studies [51–53].

To evaluate the gene expression changes associated with acute and chronic blood stage infection, DEGs at each infection timepoint were identified by comparison to the relevant baseline. Since the two baselines differed, the first acute and chronic samples were compared to the first baseline, while the second acute and chronic samples were compared to the second baseline. This analysis revealed that fewer DEGs were associated with acute infection relative to chronic infection (Fig. 4C, total genes, Fig. 4D–G). Indeed, only 36 DEGs were identified at the first acute timepoint, and zero at the second acute timepoint with a false discovery rate (FDR) of < 0.2. By contrast, 259 DEGs were identified at the first chronic timepoint, and 125 DEGs at the second chronic timepoint at this FDR. Notably, many DEGs were shared between the chronic timepoints (Fig. 4C, shared genes), suggesting substantial overlap in host response to chronic infection at both timepoints.

**Fig. 5:**
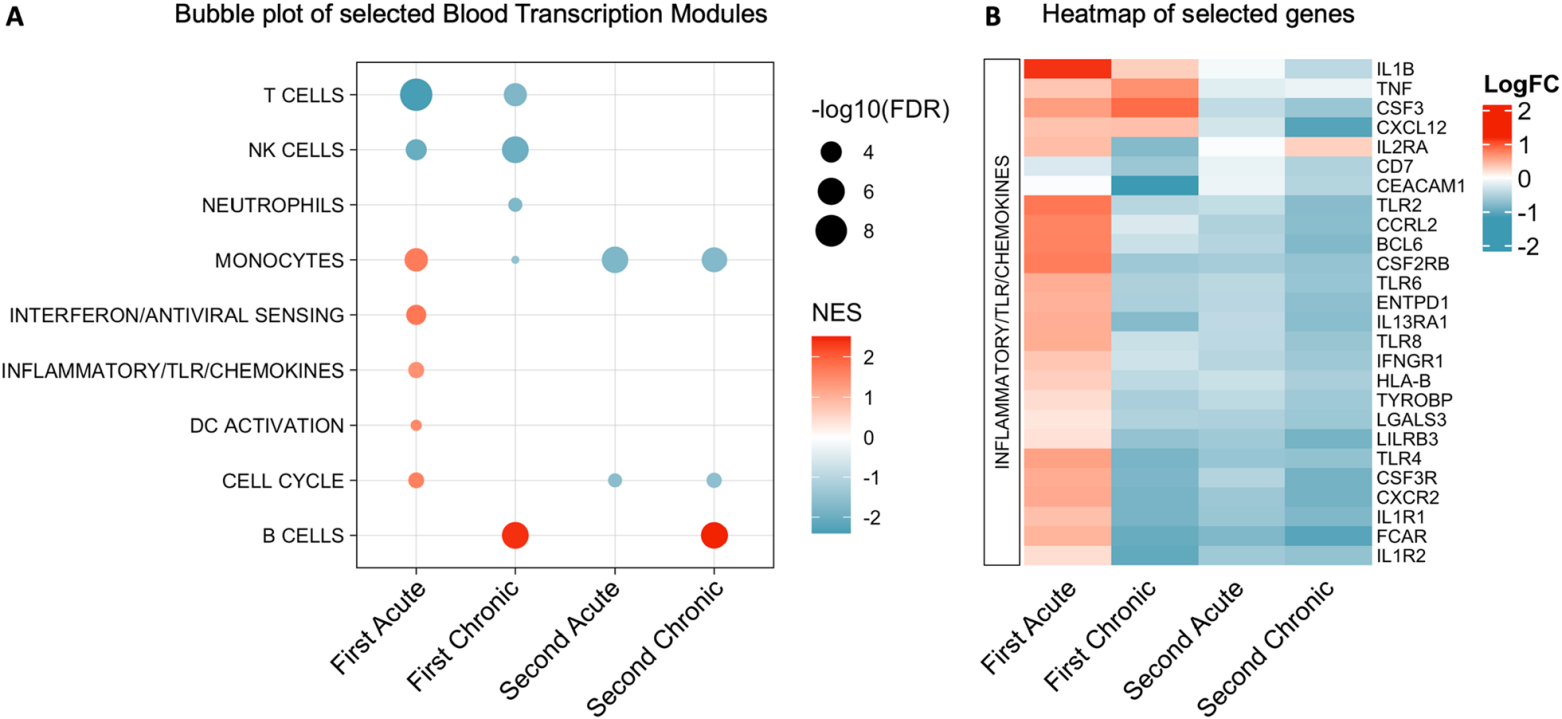
Immunological pathways associated with *P. knowlesi* infection in pig-tailed macaques. **A** Bubble plot of blood transcription modules identified by Gene Set Enrichment Analysis (GSEA). Upregulated modules shown in red and downregulated modules in blue. GSEA based on an FDR of 0.05. NES, normalized enrichment score. **B** Heatmap of genes in the inflammatory/TLR/chemokine blood transcription module that have at least one gene comparison timepoint below an FDR of 0.1. Upregulated genes in red and downregulated genes in blue. All pairwise contrasts for the first infection performed versus the first baseline, and all pairwise contrasts for the second infection performed versus the second baseline

To investigate immunological processes associated with DEGs at each of these infection timepoints, GSEA was performed using Blood Transcription Modules [42]. The first acute timepoint showed downregulation of modules for T cells and NK cells, and upregulation of modules for monocytes, interferon sensing, inflammation, dendritic cell (DC) activation and cell cycle (Fig. 5A). The upregulated modules were particularly notable, as most were uniquely observed at this timepoint, and similar gene expression changes previously described for acute blood stage malaria exposure in malaria-naïve human volunteers [54–56]. The first chronic timepoint again showed downregulation of modules for T cells and NK cells, but also downregulation of the neutrophil and monocyte modules and upregulation of the B cell module. Thus, downregulation of T and NK cells is common to both the first acute and first chronic infection timepoints, but the first acute timepoint is uniquely characterized by the upregulation of innate and inflammatory pathways, while the first chronic timepoint instead shows evidence of control of inflammation and increasing B cell responses.

Despite zero DEGs being identified for the second acute timepoint at the selected FDR, the GSEA revealed a downregulation of modules for monocytes and the cell cycle. Since these same modules were upregulated at the second baseline relative to the first baseline timepoint, this represents a shift from the altered second baseline state. Nevertheless, it was notable that the second acute timepoint did not show the same upregulation of innate and inflammatory responses as seen at the first acute timepoint, especially since control of inflammation has been associated with immune tolerance in malaria-exposed humans [51]. The second chronic timepoint likewise showed a downregulation of modules for monocytes and cell cycle, as well as upregulation of the B cell module. Thus, the second acute and chronic timepoints share downregulation of modules for monocytes, and cell cycle relative to the second baseline, but the second chronic timepoint also shows upregulation of B cell responses, similar to the first chronic infection timepoint.

Finally, to investigate inflammatory responses to *P. knowlesi* blood stage infection in PTM in more depth, a heatmap of genes associated with the inflammatory/TLR/chemokine module was made to facilitate comparison across infection timepoints. Many genes in this module were upregulated at the first acute timepoint (Fig. 5B), consistent with inflammation being identified as a defining pathway at this timepoint. Four genes from the module were also upregulated at the first chronic timepoint, including the key pro-inflammatory cytokines IL1B and TNFα. Notably however, most other genes in this module were downregulated by the first chronic timepoint, consistent with the first acute and first chronic timepoints having generally distinct immune response profiles. Following re-infection, essentially all genes in the inflammatory/TLR/chemokine module were downregulated at both the second acute and second chronic timepoints. Thus, inflammation was predominantly associated with the first acute infection timepoint, and to some extent the first chronic timepoint, while the second infection was defined by the apparent lack of inflammation. Taken together, these data suggest that modulation of inflammation, in addition to other immunological changes, is associated with the ability of PTM to tolerate and control *P. knowlesi* blood stage infection. These data further suggest that a relative lack of inflammation upon re-infection may correlate with observed development of clinical resilience in this model.

## Discussion

This exploratory study was designed to assess PTM as a potential alternative host for malaria vaccine research using *P. knowlesi* parasites. The main aims of the study were to address the two criteria most pertinent to vaccine research, namely the reliability of infection after sporozoite challenge, and the nature of the blood stage infection course. Extending from previously reported pilot data [32], this study confirmed the reliability of PkSPZ challenge in PTM, with all seven malaria-naïve animals becoming RT-PCR positive by days 6–8 p.i., and thin blood smear positive by days 11–14 p.i. The time to RT-PCR positivity was consistent with the length of the *P. knowlesi* liver stage, which is ~ 5–6 days [12]. Similarly, the time to thin blood smear positivity was consistent with data from *P. knowlesi* sporozoite challenge studies in other macaque host species (reviewed in [32]). Most importantly, the time to detection of blood stage parasites by each method was in line with prior data from rhesus macaques challenged with the same PkSPZ dose [32]. Together, this indicates that rhesus and PTM likely have equivalent susceptibility to PkSPZ challenge. Since sporozoites are typically preferred over blood stage parasites for challenge studies [57], this provides highly useful information to guide future PkSPZ challenge studies in PTM.

The ability to re-challenge animals to induce repeated *P. knowlesi* blood stage infections is another feature of the rhesus macaque model [14, 16, 17]. This study confirmed that re-infection was possible in PTM, with all three re-challenged animals becoming RT-PCR positive by day 6 p.i. However, only one re-challenged animal became transiently blood smear positive, indicating that parasitaemia was typically below the limit of detection of thin blood smears [36]. Since prior re-challenge studies in rhesus macaques relied on blood smear to monitor infection [14, 16, 17], this suggests that re-challenged PTM may develop lower blood stage burdens relative to their rhesus counterparts. However, this finding does not preclude re-challenge in PTM, but rather advocates for using sensitive diagnostic tools such as RT-PCR to monitor repeat infections. Further evaluation of the challenge and re-challenge data yields two additional insights. First, the finding that re-challenged animals become RT-PCR positive at 6 days p.i., with a similar 18S rRNA copy number to after the initial challenge, suggests that PkSPZ challenge does not induce sterilizing immunity against sporozoites or liver stages. Second, the finding that re-challenge yields different blood smear outcomes to the initial challenge suggests that infection does confer some functional anti-blood stage immunity. Both findings are preliminary and somewhat limited as only one female went on to re-challenge, but nonetheless very much in line with data from the rhesus model [14, 17], and consistent with understanding of how immunity to *Plasmodium* develops in natural human infections [58].

This preliminary study also describes several features of *P. knowlesi* infection in PTM that distinguish it from the rhesus model [11, 12, 14, 16, 17]. The daily sampling of animals for RT-PCR enabled quantitation of blood stage parasite burden over time and detailed evaluation of infection kinetics in this host. In all seven malaria-naïve animals, the blood stage infections were reproducible, with 18S rRNA copy numbers rising linearly during an initial acute infection phase, before plateauing as the infection became chronic. The initial linear rise in parasite burden is consistent with the 24-h *P. knowlesi* blood stage replication cycle [5], while the plateau indicates animals later begin to spontaneously control parasitaemia and suppress the effective parasite replication rate. This infection trajectory was accompanied by a relatively benign disease course, and at no time did animals show disease signs or need anti-malarial treatment. These findings contrast starkly with the infection outcomes reported in rhesus macaques, where *P. knowlesi* blood stage infections are typically described uncontrolled and potentially life-threatening (reviewed in [6]), and were instead much more similar to the outcomes reported in long-tailed macaques [10, 45–49]. The distinct infection outcomes in rhesus versus either the PTM or long-tailed macaque hosts in turn has very practical implications for the conduct of challenge studies. Since *P. knowlesi* can be fatal in rhesus macaques, animals must be closely monitored after challenge so that any breakthrough infections can be rapidly diagnosed and treated. Conversely, since *P. knowlesi* is apparently benign in both PTM and long-tailed macaques, animals may be less likely to experience pain and distress and thus require less intensive monitoring. Indeed, in follow-on vaccination studies, the study team has been able to reduce sampling in PTM after PkSPZ challenge to every 2 days (M. Shears, personal communication). The PTM and long-tailed macaque models may thus both offer a potential advantage over the rhesus model for challenge studies from an animal ethics perspective.

The distinct *P. knowlesi* blood stage infection outcomes in rhesus and PTM will likely also have important immunological consequences. Since PTM can safely sustain *P. knowlesi* blood stage infection for longer than rhesus macaques, they can experience a greater duration of blood stage antigen exposure, and thus may be more suited to model immune responses that take place over a longer timeframe during infection. Indeed, the finding that re-challenge leads to sub- or barely patent infections in PTM, rather than the patent infections reported in rhesus macaques [14, 17], may be due in part to differences in the length of the initial blood stage infection possible in each model and corresponding differences in the anti-blood stage humoral response. This hypothesis is supported by prior studies in rhesus and long-tailed macaques, which noted that anti-blood stage immunity was faster to develop in long-tailed macaques, and may have correlated with the longer duration of blood exposure possible in this host [17]. Another key finding of this study is thus that that both PTM and long-tailed macaques appear to share many common aspects in *P. knowlesi* disease progression and the corresponding immune response to infection, which is likely related to both macaque species being natural hosts for this parasite in the wild [3]. This ability of the PTM and long-tailed macaque models to sustain *P. knowlesi* blood stage infection for an extended time in turn may be very relevant for addressing certain vaccine research questions, e.g., how malaria infection before or during vaccination impacts the subsequent immune response. Given that candidate malaria vaccines have shown decreased immunogenicity and efficacy in malaria-endemic versus naïve populations [59], and asymptomatic malaria may not be identified and accounted for in all vaccine clinical trial designs [60], these features of *P. knowlesi* infection in the PTM and long-tailed macaque models may provide important opportunities for future research in this arena.

Finally, this pilot study used whole blood transcriptional profiling of PTM as a preliminary way to explore the host immune response to *P. knowlesi* infection. This revealed several key findings that add to understanding of infection in this host. First, *P. knowlesi* infection induced lasting immunological changes in PTM, with genes associated with the monocyte, neutrophil, cell cycle, NK cell and antigen presentation modules significantly upregulated at the second baseline relative to the first. This indicates that substantial reprogramming of innate immune cells may be occurring due to *P. knowlesi* infection and drug cure in this host, consistent with descriptions of this phenomenon in humans [52, 53]. Second, *P. knowlesi* induced distinct transcriptional changes at acute and chronic infection timepoints, which further differed between the first and second infections. The most notable of these transcriptional changes were the unique upregulation of innate and inflammatory pathways at the first acute timepoint, and the common upregulation of B cell genes at both chronic infection timepoints. Together, these data suggest the following: (1) naïve PTM mount a strong pro-inflammatory response upon initial infection, consistent with responses seen during acute blood stage malaria infection in naïve human volunteers [54–56], (2) the animals develop substantial control of inflammation by the chronic infection phases, where they also mount a robust B cell response, and (3) upon re-challenge, the animals achieve more rapid control of inflammation, consistent with response seen during acute blood stage malaria infection in previously-exposed human volunteers [56]. These findings provide insights into immune responses that correlate with *P. knowlesi* infection and the development of immune tolerance in the PTM host and offer a valuable framework to guide future systems immunology and vaccinology studies in this potential new model.

## Conclusions

This study demonstrates that experimental infection of PTM with PkSPZ results in chronic and clinically benign blood stage infections, and suggests that this new model may have distinct and complementary features to the existing rhesus model. Future studies are now planned to directly compare outcomes after PkSPZ challenge in matched cohorts of PTM and rhesus macaques to confirm the preliminary findings reported here. Overall, this new PTM model has significant potential to contribute to basic and pre-clinical malaria vaccine research.

### Supplementary Information

**Additional file 1: Figure S1. **Study design. The period of daily observations and blood collection for thin blood smears and *Plasmodium* 18S rRNA RT-PCR are indicated by bars beneath the study timeline. For cohort 2, the schedule for sedated blood draws and procedures are indicated by crosses beneath the study timeline. Animals in cohort 2 were rested for approximately two-months between the first and second PkSPZ challenge. The rest is indicated by a double slash in the timeline. PkSPZ, *Plasmodium knowlesi *sporozoites; p.i., post-infection. **Figure S2.** Principal Component analysis of whole blood gene expression data. **A** Principal Component analysis performed for all genes in the panel. **B** Principal Component analysis performed for the most variably expressed genes (420 genes under an FDR <0.2 for at least one comparison).

## Data Availability

Data related to animals, monitoring of blood stage parasite burden, monitoring of disease signs, and anti-malarial treatment will be made available from the corresponding author on reasonable request. The bulk of the whole blood gene expression data are included in this published article or its supplementary information files. Any additional data may be requested from the relevant authors (C.J.D. and T.M.T.) by reasonable request.

## Abbreviations

18S rRNA RT-PCR: 18S ribosomal RNA reverse transcription polymerase chain reaction
PkSPZ: *Plasmodium knowlesi* Sporozoites
PTM: Pig-tailed macaque
NHP: Non-human primate
DEG: Differentially expressed gene
FDR: False discovery rate
GSEA: Gene set enrichment analysis
NES: Normalized enrichment score

## References

[1] Cox-Singh J, Davis TM, Lee KS, Shamsul SS, Matusop A, Ratnam S (2008). *Plasmodium knowlesi* malaria in humans is widely distributed and potentially life threatening. Clin Infect Dis.

[2] White NJ (2008). *Plasmodium knowlesi:* the fifth human malaria parasite. Clin Infect Dis.

[3] Lee KS, Divis PC, Zakaria SK, Matusop A, Julin RA, Conway DJ (2011). *Plasmodium knowlesi:* reservoir hosts and tracking the emergence in humans and macaques. PLoS Pathog.

[4] Fungfuang W, Udom C, Tongthainan D, Kadir KA, Singh B (2020). Malaria parasites in macaques in Thailand: stump-tailed macaques (*Macaca arctoides*) are new natural hosts for *Plasmodium knowlesi*, *Plasmodium inui*, *Plasmodium coatneyi* and *Plasmodium fieldi*. Malar J.

[5] Coatney GR (1971). The primate malarias.

[6] Pasini EM, Zeeman AM, Voorberg-van der Wel A, Kocken CHM (2018). *Plasmodium knowlesi*: a relevant, versatile experimental malaria model. Parasitology.

[7] Gibbs RA, Rogers J, Katze MG, Bumgarner R, Weinstock GM, Rhesus Macaque Genome Sequencing Analysis Consortium (2007). Evolutionary and biomedical insights from the rhesus macaque genome. Science.

[8] Rogers WO, Weiss WR, Kumar A, Aguiar JC, Tine JA, Gwadz R (2002). Protection of rhesus macaques against lethal *Plasmodium knowlesi* malaria by a heterologous DNA priming and poxvirus boosting immunization regimen. Infect Immun.

[9] Hansen SG, Womack J, Scholz I, Renner A, Edgel KA, Xu G (2019). Cytomegalovirus vectors expressing *Plasmodium knowlesi* antigens induce immune responses that delay parasitemia upon sporozoite challenge. PLoS ONE.

[10] Butcher GA, Mitchell GH, Cohen S (2010). Plasmodium knowlesi infections in a small number of non-immune natural hosts (Macaca fascicularis) and in rhesus monkeys (M. mulatta). Trans R Soc Trop Med Hyg.

[11] Knowles R, Gupta BMD (1932). A study of monkey-malaria, and its experimental transmission to man. Ind Med Gaz.

[12] Garnham PC, Lainson R, Cooper W (1957). The tissue stages and sporogony of *Plasmodium knowlesi*. Trans R Soc Trop Med Hyg.

[13] Hawking F, Mellanby H, Terry RJ, Webber WA (1957). Transmission of *Plasmodium knowlesi* by *Anopheles stephensi*. Trans R Soc Trop Med Hyg.

[14] Zuckerman A (1960). Blood loss and replacement in plasmodial infections. III. *Plasmodium cynomolgi*, *Plasmodium gonderi* and *Plasmodium knowlesi* in Macaca mulatta mulatta, the rhesus monkey. J Infect Dis.

[15] Chin W, Contacos PG, Coatney GR, Kimball HR (1965). A naturally acquited quotidian-type malaria in man transferable to monkeys. Science.

[16] Brown IN, Brown KN, Hills LA (1968). Immunity to malaria: the antibody response to antigenic variation by *Plasmodium knowlesi*. Immunology.

[17] Butcher GA, Cohen S (1972). Antigenic variation and protective immunity in *Plasmodium knowlesi* malaria. Immunology.

[18] Schmidt LH, Fradkin R, Harrison J, Rossan RN (1977). Differences in the virulence of *Plasmodium knowlesi* for *Macaca irus* (*fascicularis*) of Philippine and Malayan origins. Am J Trop Med Hyg.

[19] Spangler WL, Gribble D, Abildgaard C, Harrison J (1978). *Plasmodium knowlesi* malaria in the Rhesus monkey. Vet Pathol.

[20] Chen L, Li G, Lu Y, Luo Z (2001). Histopathological changes of *Macaca mulatta* infected with *Plasmodium knowlesi*. Chin Med J (Engl).

[21] Kocken CH, Ozwara H, van der Wel A, Beetsma AL, Mwenda JM, Thomas AW (2002). *Plasmodium knowlesi* provides a rapid in vitro and in vivo transfection system that enables double-crossover gene knockout studies. Infect Immun.

[22] Colby LA, Quenee LE, Zitzow LA (2017). Considerations for infectious disease research studies using animals. Comp Med.

[23] Roodgar M, Babveyh A, Nguyen LH, Zhou W, Sinha R, Lee H (2020). Chromosome-level de novo assembly of the pig-tailed macaque genome using linked-read sequencing and HiC proximity scaffolding. Gigascience.

[24] NIH. Nonhuman primate evaluation and analysis. Part 1: analysis of future demand and supply. 2018. https://orip.nih.gov/about-orip/research-highlights/nonhuman-primate-evaluation-and-analysis-part-1-analysis-future. Accessed 21 Aug 2023.

[25] Estes JD, Wong SW, Brenchley JM (2018). Nonhuman primate models of human viral infections. Nat Rev Immunol.

[26] Batten CJ, De Rose R, Wilson KM, Agy MB, Chea S, Stratov I, Montefiori DC, Kent SJ (2006). Comparative evaluation of simian, simian-human, and human immunodeficiency virus infections in the pigtail macaque (*Macaca nemestrina*) model. AIDS Res Hum Retroviruses.

[27] Henning T, Fakile Y, Phillips C, Sweeney E, Mitchell J, Patton D (2011). Development of a pigtail macaque model of sexually transmitted infection/HIV coinfection using *Chlamydia trachomatis*, *Trichomonas vaginalis*, and SHIV(SF162P3). J Med Primatol.

[28] Cole AL, Cosgrove Sweeney Y, Lasseter AG, Gray JM, Beavis AC, Chong CF (2018). Evaluation of the pig-tailed macaque (*Macaca nemestrina*) as a model of human *Staphylococcus aureus* nasal carriage. Infect Immun.

[29] O'Connor MA, Tisoncik-Go J, Lewis TB, Miller CJ, Bratt D, Moats CR (2018). Early cellular innate immune responses drive Zika viral persistence and tissue tropism in pigtail macaques. Nat Commun.

[30] Melton A, Doyle-Meyers LA, Blair RV, Midkiff C, Melton HJ, Russell-Lodrigue K (2021). The pigtail macaque (*Macaca nemestrina*) model of COVID-19 reproduces diverse clinical outcomes and reveals new and complex signatures of disease. PLoS Pathog.

[31] Fooden J (1994). Malaria in macaques. Int J Primatol.

[32] Chakravarty S, Shears MJ, James ER, Rai U, Kc N, Conteh S (2022). Efficient infection of non-human primates with purified, cryopreserved *Plasmodium knowlesi* sporozoites. Malar J.

[33] Arnegard ME, Whitten LA, Hunter C, Clayton JA (2020). Sex as a biological variable: a 5-year progress report and call to action. J Womens Health (Larchmt).

[34] Sullivan JS, Morris CL, Richardson BB, Galland GG, Sullivan JJ, Collins WE (1996). Sporozoite transmission of three strains of *Plasmodium knowlesi* to *Aotus* and *Saimiri* monkeys. J Parasitol.

[35] Shears MJ, Seilie AM, Kim Lee Sim B, Hoffman SL, Murphy SC (2020). Quantification of *Plasmodium knowlesi* versus *Plasmodium falciparum* in the rhesus liver: implications for malaria vaccine studies in rhesus models. Malar J.

[36] Zimmerman PA, Howes RE (2015). Malaria diagnosis for malaria elimination. Curr Opin Infect Dis.

[37] Murphy SC, Daza G, Chang M, Coombs R (2012). Laser cutting eliminates nucleic acid cross-contamination in dried-blood-spot processing. J Clin Microbiol.

[38] Seilie AM, Chang M, Hanron AE, Billman ZP, Stone BC, Zhou K (2019). Beyond blood smears: qualification of *Plasmodium* 18S rRNA as a biomarker for controlled human malaria infections. Am J Trop Med Hyg.

[39] Ritchie ME, Phipson B, Wu D, Hu Y, Law CW, Shi W (2015). limma powers differential expression analyses for RNA-sequencing and microarray studies. Nucleic Acids Res.

[40] Lex A, Gehlenborg N, Strobelt H, Vuillemot R, Pfister H (2014). UpSet: visualization of intersecting sets. IEEE Trans Vis Comput Graph.

[41] Conway JR, Lex A, Gehlenborg N (2017). UpSetR: an R package for the visualization of intersecting sets and their properties. Bioinformatics.

[42] Li S, Rouphael N, Duraisingham S, Romero-Steiner S, Presnell S, Davis C (2014). Molecular signatures of antibody responses derived from a systems biology study of five human vaccines. Nat Immunol.

[43] Subramanian A, Tamayo P, Mootha VK, Mukherjee S, Ebert BL, Gillette MA (2005). Gene set enrichment analysis: a knowledge-based approach for interpreting genome-wide expression profiles. Proc Natl Acad Sci USA.

[44] Collins WE (2012). *Plasmodium knowlesi*: a malaria parasite of monkeys and humans. Annu Rev Entomol.

[45] Collins WE, Skinner JC, Broderson JR, Filipski VK, Morris CM, Stanfill PS (1992). Susceptibility of *Macaca fascicularis* monkeys from Mauritius to different species of *Plasmodium*. J Parasitol.

[46] Butcher GA (1996). Models for malaria: nature knows best. Parasitol Today.

[47] Anderios F, Noorrain A, Vythilingam I (2010). In vivo study of human *Plasmodium knowlesi* in *Macaca fascicularis*. Exp Parasitol.

[48] Barber BE, Russell B, Grigg MJ, Zhang R, William T, Amir A (2018). Reduced red blood cell deformability in *Plasmodium knowlesi* malaria. Blood Adv.

[49] Peterson MS, Joyner CJ, Brady JA, Wood JS, Cabrera-Mora M, Saney CL (2021). Clinical recovery of *Macaca fascicularis* infected with *Plasmodium knowlesi*. Malar J.

[50] Schnitzer B, Sodeman TM, Mead ML, Contacos PG (1973). An ultrastructural study of the red pulp of the spleen in malaria. Blood.

[51] Portugal S, Moebius J, Skinner J, Doumbo S, Doumtabe D, Kone Y (2014). Exposure-dependent control of malaria-induced inflammation in children. PLoS Pathog.

[52] Mpina M, Maurice NJ, Yajima M, Slichter CK, Miller HW, Dutta M (2017). Controlled human malaria infection leads to long-lasting changes in innate and innate-like lymphocyte populations. J Immunol.

[53] Bediako Y, Adams R, Reid AJ, Valletta JJ, Ndungu FM, Sodenkamp J (2019). Repeated clinical malaria episodes are associated with modification of the immune system in children. BMC Med.

[54] Tran TM, Jones MB, Ongoiba A, Bijker EM, Schats R, Venepally P (2016). Transcriptomic evidence for modulation of host inflammatory responses during febrile *Plasmodium falciparum* malaria. Sci Rep.

[55] Tran TM, Bijker EM, Haks MC, Ottenhoff THM, Visser L, Schats R (2019). Whole-blood transcriptomic signatures induced during immunization by chloroquine prophylaxis and *Plasmodium falciparum* sporozoites. Sci Rep.

[56] de Jong SE, van Unen V, Manurung MD, Stam KA, Goeman JJ, Jochems SP (2021). Systems analysis and controlled malaria infection in Europeans and Africans elucidate naturally acquired immunity. Nat Immunol.

[57] Spring M, Polhemus M, Ockenhouse C (2014). Controlled human malaria infection. J Infect Dis.

[58] Hafalla JC, Silvie O, Matuschewski K (2011). Cell biology and immunology of malaria. Immunol Rev.

[59] Mo AXY, Pesce J, Augustine AD, Bodmer JL, Breen J, Leitner W (2020). Understanding vaccine-elicited protective immunity against pre-erythrocytic stage malaria in endemic regions. Vaccine.

[60] Owalla TJ, Hergott DEB, Seilie AM, Staubus W, Chavtur C, Chang M (2022). Rethinking detection of pre-existing and intervening *Plasmodium* infections in malaria clinical trials. Front Immunol.

