# Supplementary material for: Plasmodium knowlesi in pig-tailed macaques: a potential new model for malaria vaccine research
Source: Malar J. 2023 Dec 13;22:379. doi: 10.1186/s12936-023-04788-9 (PMC10720125; doi:10.1186/s12936-023-04788-9)
Supplement: Supplementary file 1 — Additional file 1: Figure S1. Study design. The period of daily observations and blood collection for thin blood smears and Plasmodium 18S rRNA RT-PCR are indicated by bars beneath the study timeline. For cohort 2, the schedule for sedated blood draws and procedures are indicated by crosses beneath the study timeline. Animals in cohort 2 were rested for approximately two-months between the first and second PkSPZ challenge. The rest is indicated by a double slash in the timeline. PkSPZ, Plasmodium knowlesi sporozoites; p.i., post-infection. Figure S2. Principal Component analysis of whole blood gene expression data. A Principal Component analysis performed for all genes in the panel. B Principal Component analysis performed for the most variably expressed genes (420 genes under an FDR <0.2 for at least one comparison). [file 12936_2023_4788_MOESM1_ESM.docx]

**Supplementary Information**

**
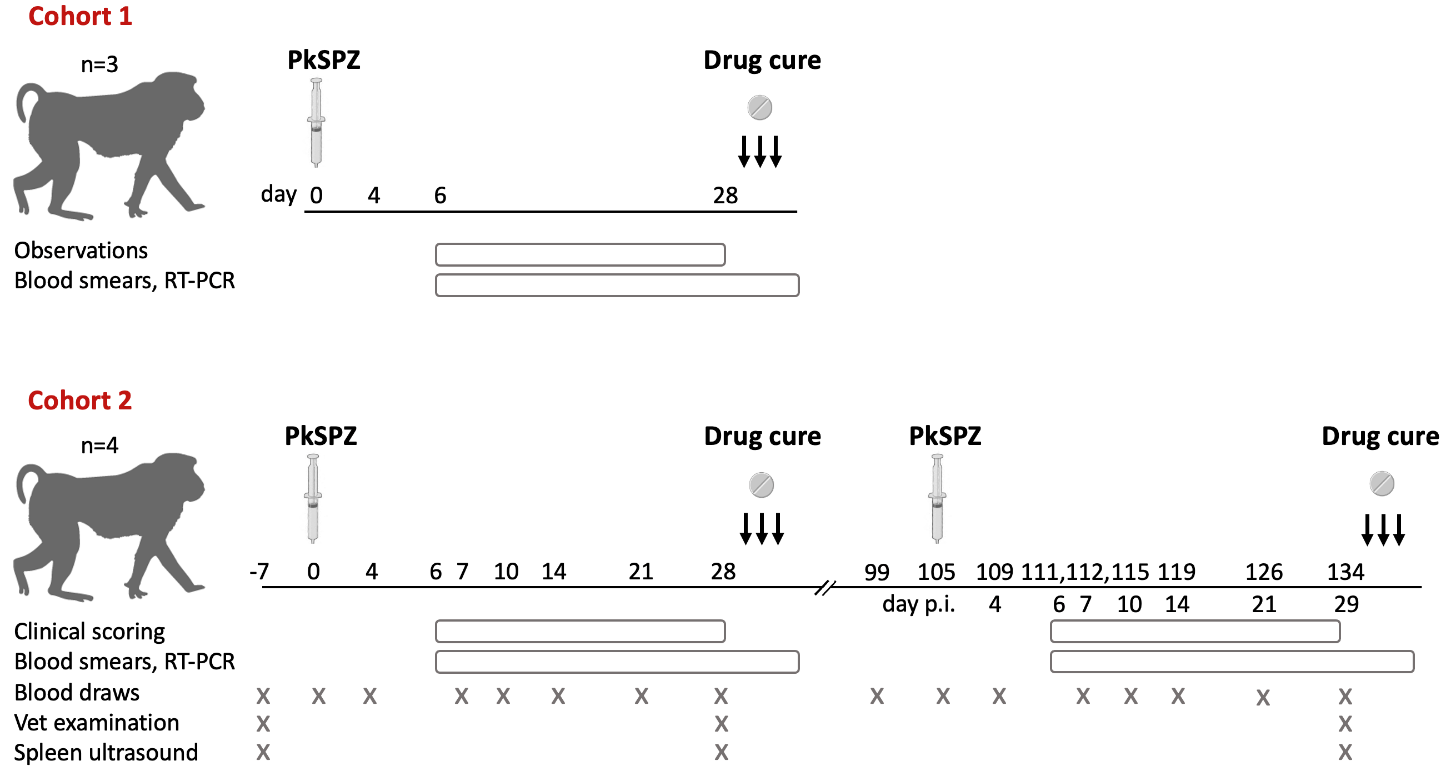
Supplementary Figure 1. Study design.** The period of daily observations and blood collection for thin blood smears and *Plasmodium* 18S rRNA RT-PCR are indicated by bars beneath the study timeline. For cohort 2, the schedule for sedated blood draws and procedures are indicated by crosses beneath the study timeline. Animals in cohort 2 were rested for approximately two-months between the first and second PkSPZ challenge. The rest is indicated by a double slash in the timeline. Abbreviations: PkSPZ; *Plasmodium knowlesi* sporozoites; p.i., post-infection.

**Supplementary Figure 2:** Principal Component analysis of whole blood gene expression data. **A**. Principal Component analysis performed for all genes in the panel. **B**. Principal Component analysis performed for the most variably expressed genes (420 genes under an FDR <0.2 for at least one comparison).


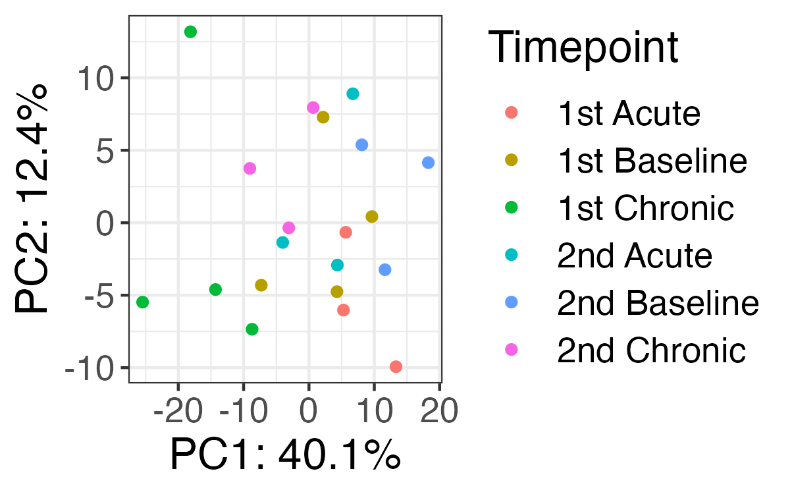

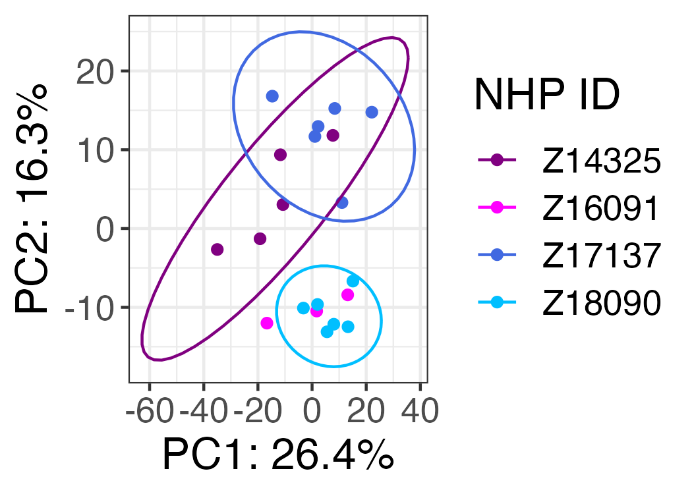


**A**

**B**
